# Efficacy and Safety of Non-cross-Linked Hyaluronic Acid Injections for Facial Skin Biorevitalization: A Single-Center, Open-Label, Single-Arm, Uncontrolled, Post-marketing Study

**DOI:** 10.7759/cureus.90005

**Published:** 2025-08-13

**Authors:** Stefano Chahine, Barbara Marozzi, Alessandro Valle, Laura Michellini, Tiziana Lazzari

**Affiliations:** 1 Pharmacy, Phitogen Beauty Labs SpA, San Benedetto del Tronto, ITA; 2 Dermatology, CDS (Casa della Salute) SpA, Genova, ITA; 3 Pharmacology, Latis CRO Srl, Genova, ITA

**Keywords:** aesthetic improvement, biorevitalization, face, hyaluronic acid, skin capacitance

## Abstract

Background: Biorevitalization is a minimally invasive cosmetic procedure that involves the targeted intradermal injection of bioactive substances designed to enrich the skin and stimulate its natural regenerative capabilities. The aim of this single-center, open-label, single-arm, uncontrolled, post-marketing study was to examine the effectiveness and safety of biostimulatory injections using a proprietary, sterile gel containing non-cross-linked hyaluronic acid (Foliage Hydrofil^®^) for facial skin rejuvenation.

Methods: The trial was registered with ClinicalTrials.gov (NCT07010549). The intended target population comprised adults with mild-to-moderate facial photoaging and Fitzpatrick skin types III-IV, without active inflammatory dermatoses or recent aesthetic interventions. Twenty-four Caucasian subjects (21 women, three men; mean age: 44 ± 7.1 years) received three intradermal injections of the study product at three-week intervals. The primary endpoint was the change from baseline in skin capacitance, an objective measure of hydration, assessed via corneometry. Secondary endpoints included investigator and subject assessments of aesthetic improvement, while safety was evaluated through continuous adverse event monitoring.

Results: The mean skin capacitance was 23.1 arbitrary units (a.u.) at baseline and increased to 33.2 a.u. at the end of the study, indicating a significant improvement in skin hydration (p < 0.05). Secondary outcomes showed moderate improvements in skin texture and tone, and high levels of participant satisfaction with the treatment. No adverse events were reported.

Conclusions: This study demonstrated that the tested non-cross-linked hyaluronic acid gel safely improves skin hydration and patient satisfaction in facial biorevitalization. Although promising, these findings are constrained by the study's uncontrolled, small-scale design; therefore, larger randomized controlled trials are required to confirm these outcomes and evaluate their long-term durability.

## Introduction

Skin aging results from a complex interplay between extrinsic and intrinsic factors, manifesting as visible changes, including fine lines, wrinkles, coarser skin texture, and reduced cutaneous firmness [1,2]. A key characteristic of this process is decreased hydration within the stratum corneum (SC), which plays a critical role in the skin's overall health and appearance [3]. In recent years, skin capacitance has emerged as a crucial measure of cutaneous hydration, reflecting the SC's ability to hold an electrical charge [4,5]. The principle underlying capacitance measurement is based on the observation that skin's dielectric properties vary with hydration level, with well-hydrated skin exhibiting greater capacity for electrical charge storage [6]. Skin capacitance decreases significantly with advancing age, correlating with the increased cutaneous dryness characteristic of aging skin [7]. Therefore, maintaining elevated capacitance levels is essential for preserving skin elasticity, functional integrity, and aesthetic appeal.

Hyaluronic acid (HA) is a pivotal molecule in maintaining skin hydration due to its remarkable water retention capacity [8,9]. Notably, evidence demonstrates that a single gram of HA can absorb up to six liters of water, highlighting its critical role in regulating cutaneous moisture [10]. However, the natural aging process leads to a significant reduction in endogenous HA concentration within the skin. A landmark study revealed that by age 75, individuals retain less than 25% of the HA content found in 19-year-olds, resulting in markedly decreased moisture retention capacity and compromised repair mechanisms [11]. This reduction in HA content increases skin susceptibility to environmental damage, underscoring HA's importance in maintaining barrier function [12]. Additionally, HA plays a critical role in modulating inflammatory pathways, helping prevent collagen and elastin degradation while supporting skin resilience [13]. These age-related alterations in HA dynamics substantially contribute to skin dryness, wrinkle formation, and loss of elasticity, ultimately affecting aesthetic appearance [12].

Biorevitalization is a non-surgical, minimally invasive cosmetic procedure involving targeted intradermal injection of bioactive substances designed to enrich the skin and stimulate natural regenerative capabilities [14]. By utilizing the unique characteristics of non-cross-linked HA [15], biorevitalization enhances skin hydration while stimulating fibroblasts [16], thereby promoting collagen and elastin production essential for youthful cutaneous appearance. Building on these principles, the present study examined the effectiveness and safety of biostimulatory injections using a proprietary, sterile gel containing non-cross-linked HA for facial skin rejuvenation. A comprehensive approach was adopted, incorporating skin capacitance measurements and a dual-evaluation system in which both investigators and participants assessed the observed aesthetic improvements.

## Materials and methods

Ethics

The trial was registered with ClinicalTrials.gov (NCT07010549). The investigation was carried out at a single aesthetic medicine center (Casa della Salute SpA, Genoa, Italy), approved by the local ethics committee (CER Liguria; approval number: 75/2022; approval date: June 20, 2022), and conducted in accordance with the Declaration of Helsinki. All participants provided written informed consent prior to enrollment.

Study design and participants

This was a single-center, open-label, single-arm, uncontrolled, post-marketing study, which started in September 2022 and concluded in February 2023. Eligibility was based on the following criteria: (1) Caucasian men and women aged 30-55 years with a Fitzpatrick skin type III or IV, who were seeking aesthetic treatments to improve their facial appearance; (2) individuals showing visible signs of aging on their facial skin; and (3) participants with baseline corneometer skin capacitance measurements below 80 arbitrary units (a.u.) in the facial areas to be treated. In general, optimal hydration rates evaluated with the corneometer range between 60 and 80 a.u., although values equal to or greater than 40-45 a.u. are considered indicative of sufficient hydration [17].

Exclusion criteria comprised individuals with Fitzpatrick skin types V or VI; pregnant or breastfeeding women; subjects with known allergies or hypersensitivity to the components of the study product; individuals with extensive photodamage or severe skin aging; those with active skin diseases or conditions such as infections, perioral dermatitis, seborrheic eczema, and rosacea affecting the face, hands, or décolletage; individuals with immune system disorders; subjects with a history of or active collagen vascular diseases (e.g., systemic lupus erythematosus, rheumatoid arthritis, and cutaneous or systemic sclerosis); individuals with a history of cancerous or precancerous lesions on the face, neck, and décolletage; and those with tattoos on the skin surrounding the treatment site. Additionally, individuals were excluded if they had participated in another clinical study within 30 days prior to the initial visit or planned to participate in another investigation during the course of this study; were undergoing or planning to undergo a weight reduction program during the study period; had a known history of drug or alcohol abuse within the six months preceding the baseline visit; had known hypersensitivity to any components of anesthetic creams; used nicotine during the study or had ceased its use within 12 months prior to the first visit; or had any medical condition that, in the opinion of the investigator, rendered them unsuitable for inclusion. Other exclusion criteria comprised circumstances that, in the investigator's judgment, would likely lead to participants being unreliable, unable to attend follow-up visits, or engaging in protocol violations. Finally, potential participants were deemed ineligible if they had undergone any of the following treatments within the specified time frames prior to the baseline visit: (1) non-permanent filler or botulinum toxin treatments, aesthetic surgeries, laser therapies, mesotherapy, or any facial, hand, or décolletage peeling within the last 12 months; (2) injectable revitalization treatments within the last 12 months; (3) retinoic acid use within the last six months; and (4) chemotherapy, immunosuppressive drugs, corticosteroids, thrombolytics, anticoagulants, or platelet aggregation inhibitors within the last two weeks. A history of treatment with permanent fillers was a disqualifying criterion. Eligibility restrictions were designed a priori to minimize confounders known to influence corneometric measurements and subjective aesthetic assessments, thereby improving the interpretability of efficacy and safety outcomes.

Study product

The study product, Foliage Hydrofil^®^ (Phitogen Holding S.p.A., San Benedetto del Tronto, Italy), is a CE-marked, class III medical device designed to enhance skin hydration and address superficial skin imperfections. The device consists of 2 mL injectable vials containing a sterile, non-pyrogenic, resorbable gel. Each vial contains 32 mg of non-cross-linked HA sodium salt derived through bacterial fermentation from non-animal sources, with molecular weights (200, 500, and 1000 KDa) comparable to human HA. The formulation is completed with phosphate-buffered saline solution to achieve a total volume of 2 mL.

Procedures

**Figure 1:**
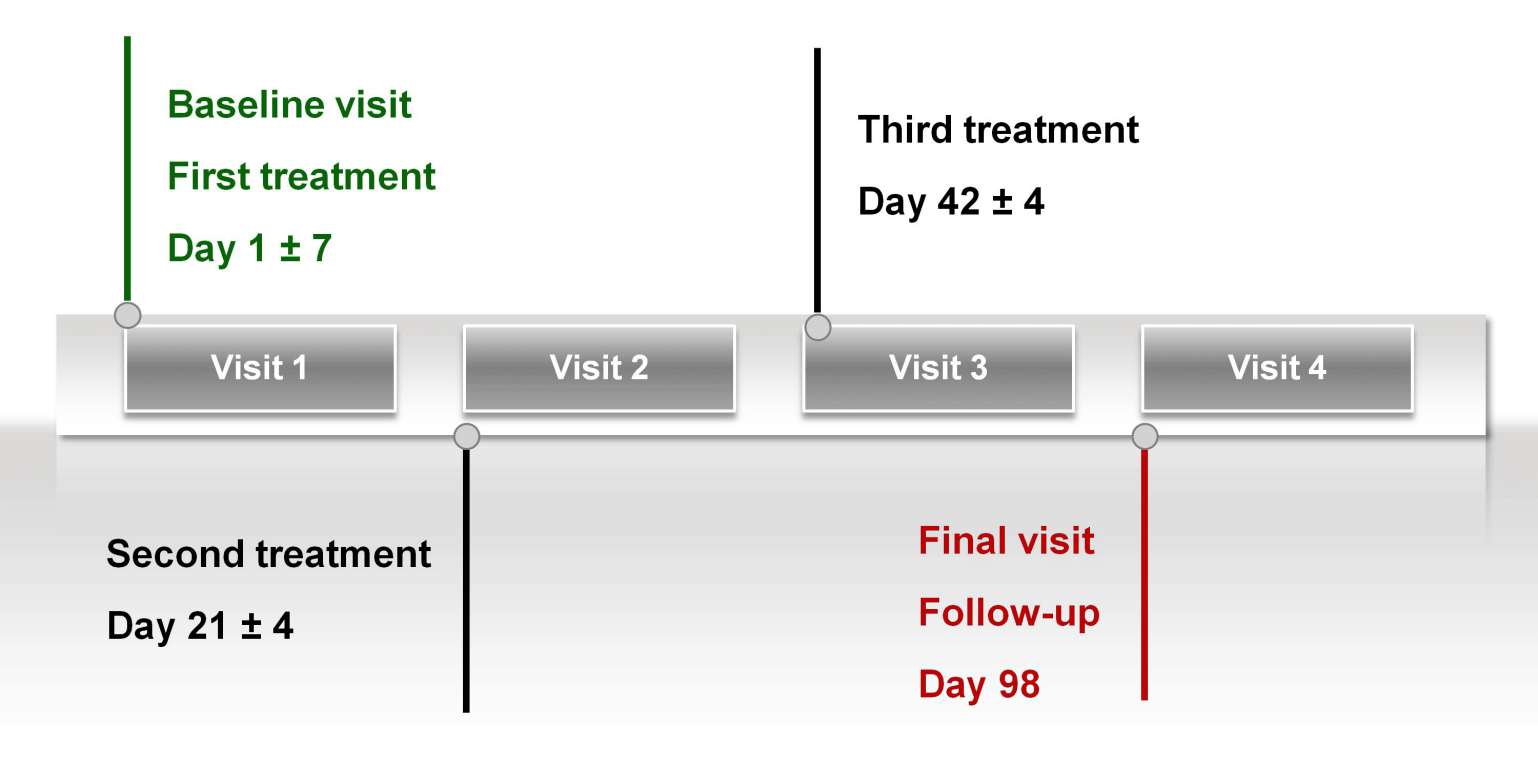
Timeline of the four study visits.

All participants received three intradermal injections of the study product to the facial region (lateral canthal area and cheek) using a 30 G × 4 mm needle, with treatments administered at three-week intervals across separate sessions. Multiple microinjections were performed at 1-2 cm intervals, with up to 2 mL total volume per session according to the investigator's judgment. Injection sites were disinfected prior to treatment, and local anesthetic cream was applied at the investigator's discretion. The study protocol consisted of four distinct visits (Figure 1). Visit 1 served to determine participant eligibility and administer the initial injection. Visits 2 and 3 were scheduled at 21 ± 4 days and 42 ± 4 days after the initial session, respectively, for subsequent treatment sessions. The final assessment (visit 4) occurred 98 ± 7 days after study initiation.

Primary endpoint

The primary efficacy endpoint was the change in skin capacitance from visit 1 to visit 4, measured non-invasively using a corneometer (APM PRO 100; Aram Huvis Co., Ltd., Seongnam, South Korea) in treated facial areas under controlled environmental conditions. Prior to measurement, participants underwent a 30-minute acclimatization period in a controlled environment maintained at 20-22°C temperature and 45-55% relative humidity to ensure measurement standardization and reduce environmental variability. The instrument measures capacitance values that correlate with stratum corneum hydration to an approximate depth of 10−20 μm by briefly pressing a probe tip containing a specialized glass element to the skin surface. The measurement area was approximately 49 mm^2^, and results were reported in arbitrary units. Participants demonstrating an improvement of at least 7.5 a.u. in skin capacitance compared to baseline were classified as responders.

Secondary endpoints

Secondary efficacy endpoints included the following: (1) evaluating product effectiveness in improving skin capacitance at visits 2 and 3; (2) assessing improvements in skin texture, fine lines, skin tone, and wrinkles at visits 2-4; (3) examining aesthetic changes as perceived by both investigators and subjects; and (4) evaluating subjects' overall treatment satisfaction at visits 2-4. Skin texture and fine line improvement in treated facial areas were assessed using a three-point Likert scale: 1 (poor), 2 (good), and 3 (very good). Skin tone was evaluated using a four-point scale: 1 (poor), 2 (sufficient), 3 (moderate), and 4 (good). Wrinkle assessment utilized a five-point scale ranging from 1 (absent) to 5 (extreme). Subjects were classified as responders when the investigator's assessment of skin texture, fine lines, and skin tone demonstrated improvement of at least one category from visit 1. For wrinkles, subjects were considered responders when the investigator's evaluation showed a reduction of at least two grades from visit 1, or when severity decreased from 2 (slight) to 1 (absent). Investigator assessment of aesthetic changes was conducted using the Global Aesthetic Improvement Scale (GAIS) [18], a five-point Likert scale ranging from 1 (worse than before treatment) to 5 (optimal improvement). Subjective aesthetic changes were evaluated using the Patient's Aesthetic Improvement Scale (PAIS) [14], also a five-point Likert scale with descriptors from 1 (worse) to 5 (very much improved). Overall treatment satisfaction was assessed using a five-point scale ranging from 1 (very satisfied) to 5 (very unsatisfied).

Safety evaluation

The safety evaluation included a comprehensive assessment of adverse events (AEs) occurring during the study, documenting the number, type, seriousness, severity, and relationship to study treatment. Local tolerability at injection sites was also determined.

Sample size determination

We hypothesized an average increase in skin capacitance of 7.5 a.u., measured by corneometry, from visit 1 to visit 4, with an anticipated standard deviation of 8 a.u. Sample size calculation indicated that 20 subjects would be required to detect this difference with 95% power and an alpha level of 0.05. Accounting for an anticipated 20% dropout rate, the recruitment target was set at 24 subjects.

Data analysis

The analysis comprised two distinct populations: the safety analysis set (SAF) and the full analysis set (FAS). The SAF comprised all 24 enrolled participants who provided informed consent and received at least one study product administration. The FAS included 23 subjects, excluding one participant who lacked post-visit 1 assessments. Categorical data were summarized as frequencies and percentages, while continuous data were presented as descriptive statistics, including mean, standard deviation (SD), median, and range. Efficacy parameters across visits were compared using the Wilcoxon signed-rank test. Statistical analyses were performed using SAS version 9.4 (SAS Institute Inc., Cary, NC), with two-tailed p-values < 0.05 considered statistically significant.

## Results

Participant characteristics

Table 1 summarizes the demographics and baseline characteristics of the 24 study participants. One subject withdrew consent between visit 1 and visit 2; therefore, 23 subjects comprised the FAS for efficacy endpoints, whereas all 24 were included in the SAF.

**Table 1:** Participant demographics and baseline characteristics. Data are presented as count (%) or mean ± standard deviation and range, as appropriate.

Characteristic	Value
Number of participants	24
Sex, n (%)	
Male	3 (12.5%)
Female	21 (87.5%)
Age, years (mean ± standard deviation, range)	44.0 ± 7.1 (32–54)
Fitzpatrick skin type, n (%)	
Type III	20 (83.3%)
Type IV	4 (16.7%)

Primary efficacy endpoint

In the FAS population (n = 23), mean skin capacitance increased from 23.1 ± 9.6 a.u. at visit 1 to 33.2 ± 9.3 a.u. at visit 4 (Table 2). The mean change was 10.0 ± 13.7 a.u. (median: 7; range: -15 to 38), which was statistically significant (Wilcoxon signed-rank test: Z = -3.25, p = 0.001; significance defined as p < 0.05). At visit 4, 11 of 23 participants (47.8%) met the pre-specified response criterion (≥7.5 a.u. increase from baseline).

**Table 2:** Skin capacitance at baseline (visit 1) and after treatment (visit 4) in the full analysis set (FAS) population. Data are shown as mean ± standard deviation, median (range), or n (%), as appropriate. The Wilcoxon signed-rank test was applied for statistical analysis. Significance threshold set at p < 0.05.

Timepoint	Mean ± SD (a.u.)	Median (range)	Responders, n (%)	Z-value	p-value
Visit 1 (baseline)	23.1 ± 9.6	—	—	—	—
Visit 4 (final)	33.2 ± 9.3	—	11 (47.8%)	-3.25	0.001
Change (V4–V1)	10.0 ± 13.7	7 (-15 to 38)	—	—	—

Skin capacitance over time

Mean skin capacitance increased progressively at each visit, with significant differences from baseline at all follow-up visits (Table 3).

**Table 3:** Skin capacitance and response rates at each study visit in the full analysis set (FAS) population. Data are shown as mean ± standard deviation or n (%), as appropriate. Statistical comparisons versus baseline (visit 1) by the Wilcoxon signed-rank test. Significance threshold set at p < 0.05.

Visit	Mean ± SD (a.u.)	Range (a.u.)	Responders, n (%)	Z-value versus visit 1	P-value
Visit 1	23.1 ± 9.6	7–43	—	—	—
Visit 2	33.0 ± 6.6	16–43	16 (69.6%)	-3.19	0.001
Visit 3	34.8 ± 7.5	15–49	16 (69.6%)	-3.14	0.001
Visit 4	33.2 ± 9.3	20–57	11 (47.8%)	-3.25	0.001

Secondary endpoints

Assessment of secondary efficacy endpoints is summarized in Table 4. Improvements were observed over time in skin texture, fine lines, and skin tone, though responder rates remained low for some measures. No participants were classified as responders for wrinkles at any visit. The proportion of participants judged as improved by the investigator (GAIS) was 87.0% at visit 2, 78.3% at visit 3, and reached 100% at visit 4. Patient-reported improvements (PAIS) were maintained at roughly two-thirds for all post-baseline visits. Satisfaction with the study product was high at study end, with 18/23 (78.3%) “Satisfied” and 5/23 (21.7%) “Very Much Satisfied.”

**Table 4:** Responder rates and subjective improvement indices by visit in the full analysis set (FAS) population. Data are reported as n (%). Definitions of response as per protocol criteria. GAIS: Global Aesthetic Improvement Scale; PAIS: Patient's Aesthetic Improvement Scale.

Endpoint	Visit 2	Visit 3	Visit 4
Skin texture responders	0 (0%)	1 (4.3%)	4 (17.4%)
Fine lines responders	0 (0%)	0 (0%)	1 (4.3%)
Skin tone responders	3 (13.0%)	3 (13.0%)	5 (21.7%)
Wrinkle responders	0 (0%)	0 (0%)	0 (0%)
GAIS improved	20 (87.0%)	18 (78.3%)	23 (100%)
PAIS improved	16 (69.6%)	15 (65.2%)	16 (69.6%)
Participants satisfied with the study product	—	—	18 (78.3%)
Participants very satisfied with the study product	—	—	5 (21.7%)

Safety

The study product exhibited a favorable safety profile and was consistently well-tolerated throughout the entire investigation. Within the SAF population, no AEs were documented. This included the absence of pain, bruising, local inflammation, swelling, infection, hyperpigmentation, edema, allergic reactions, as well as the lack of seroma, granuloma, hematoma, or capsule formation.

## Discussion

In the field of aesthetic medicine, HA is distinguished by its hydrophilic, viscoelastic, and biocompatible properties, making it a versatile molecule with broad clinical applications [11]. Cross-linked, hyaluronidase-resistant HA is extensively used in dermal filler formulations for volumetric restoration and contour enhancement [19]. In contrast, non-cross-linked HA has emerged as a key component in biorevitalization procedures for aging skin [16], improving hydration and stimulating fibroblast activity to facilitate endogenous collagen and elastin production [20]. This open-label study evaluated the efficacy and safety of a proprietary non-cross-linked HA medical device for intradermal multi-injection biorevitalization. Our primary objective was to determine the treatment effect on facial skin capacitance, a pivotal measure of stratum corneum hydration [6,7]. Additionally, we assessed post-procedural aesthetic improvements as evaluated by both investigators and participants.

The study yielded three principal findings. First, regarding stratum corneum hydration, we observed a significant increase in cutaneous capacitance from the second study visit onward, which persisted until study completion. By visit 2, 69.6% of participants achieved the desired treatment outcome (increase of at least 7.5 a.u. in skin capacitance from baseline). This substantial response rate was maintained, with 47.8% of participants still meeting response criteria at final follow-up. These findings demonstrate the sustained hydrating benefits of the tested product. Second, regarding post-procedural aesthetic improvements, a moderate proportion of participants showed enhancements at study end compared to baseline. Specifically, 17.4% of participants experienced improved skin texture, and 21.7% demonstrated enhanced skin tone, as assessed by investigators. The GAIS questionnaire revealed statistically significant aesthetic improvements at each follow-up visit compared to baseline, indicating high investigator satisfaction. PAIS data further supported these outcomes, reflecting positive patient treatment evaluations. All participants reported satisfaction with biorevitalization results using the study product, with 20% expressing high satisfaction. Finally, non-cross-linked HA gel injections demonstrated excellent tolerability, with no significant adverse events reported in the safety analysis population.

Our findings on the study product's effectiveness in enhancing biophysical (capacitance) and clinical (skin texture and tone) parameters are in accordance with existing biorevitalization literature. Accordingly, Duteil et al. [16] recently conducted a comparative study examining the effects of non-cross-linked HA injection on facial aging markers using saline solution as a placebo. Results showed significant improvements in skin hydration, wrinkle reduction, increased firmness, and enhanced radiance after three treatment sessions [16]. Superficial skin hydration measurements demonstrated a pronounced 35% increase from day one to day 53 on the treated side. Cheng et al. [21] documented a 9% increase in superficial skin hydration three months after five weekly pneumatic non-cross-linked HA injection sessions. In addition, Lee et al. [22] found a modest but statistically significant 6% increase in skin hydration six weeks after three injection sessions.

In our study, the full analysis set population exhibited a significant increase in mean skin capacitance from 23.1 a.u. at baseline to 33.2 a.u. at the fourth visit, representing a 43.7% increase. While the mechanisms underlying this enhanced response compared to previous non-cross-linked HA biorevitalization studies [16,21,22] remain unclear, we hypothesize that the study product's unique formulation, containing HA with molecular weights similar to endogenous human HA, may be a key contributing factor. The instrumental findings supporting the product's beneficial impact on skin hydration were further validated through enhanced post-procedural aesthetic appearance, as reported by both investigators (GAIS) and participants (PAIS). GAIS responses revealed that the proportion of subjects showing improvement ranged from 78.3% at the third visit to 100% by the fourth visit. These findings are consistent with those reported by Duteil et al. [16], who demonstrated that non-cross-linked HA injections resulted in improved or markedly improved aesthetic appearance in 82% of their study cohort. Safety assessments revealed no adverse events, confirming excellent tolerability of the study product. This safety profile was superior to those previously reported for similar products. For instance, Baspeyras et al. [23] reported 50 mild-to-moderate adverse events among 57 participants in their safety evaluation of non-reticulated HA-based mesotherapy. Similarly, Duteil et al. [16] found that transient inflammatory reactions, hematoma, edema, papules, and erythema were significantly more prevalent on the non-cross-linked HA-treated side (46.8%) compared to the placebo-treated side (25.7%). These results suggest the comparatively superior safety profile of our study product.

Albeit promising, the interpretation of our findings is subject to several important limitations that must be considered when evaluating their clinical significance. The most fundamental caveat stems from the single-arm, open-label design. Without a randomized controlled comparator arm, we cannot provide direct comparative efficacy data against other non-cross-linked HA-based injectable products or establish definitive causal relationships between treatment and observed outcomes. While this open-label approach was chosen to reflect real-world clinical practice, it inherently introduces bias risk since neither the investigators nor the participants were blinded to treatment. Although blinding is frequently challenging in aesthetic treatment evaluations, the reliance on unblinded assessments by treating investigators represents a significant methodological constraint. Additionally, the stringent eligibility criteria, while reducing variability and strengthening effect attribution in our single-arm design, substantially limit external generalizability. Our findings are most applicable to adults with mild-to-moderate photoaging and Fitzpatrick skin types III-IV, excluding those with active inflammatory skin disease or recent aesthetic procedures. To enhance external validity and facilitate replication across more diverse clinical settings, future studies should expand eligibility criteria to include broader Fitzpatrick types, age ranges, improved sex balance, and participants with controlled comorbidities or recent aesthetic histories. Several factors also constrain our ability to draw definitive conclusions about clinical efficacy. The primary endpoint of skin capacitance reflects superficial hydration but may not directly translate to meaningful aesthetic improvement without corroboration from blinded, clinically anchored measures. Moreover, while statistically significant improvements in hydration were observed, the actual improvements in skin texture and tone were limited, and wrinkle reduction was negligible in the short-term follow-up period. Additionally, the focus on facial treatments precluded performing skin biopsies, which would have provided direct evaluation of post-procedural collagen and elastin fiber deposition. The positive GAIS/PAIS ratings, while encouraging, should be interpreted cautiously given the unblinded assessment methodology and the modest nature of the observed clinical changes. Furthermore, the small single-center cohort limits both statistical power and generalizability of findings. The relatively short follow-up period constrains our understanding of treatment durability, and the lack of predefined clinically meaningful thresholds for improvement makes it difficult to distinguish between statistically significant and clinically relevant changes. While our results suggest potential therapeutic benefit, they should be viewed as exploratory signals requiring confirmation through controlled trials with blinding, predefined clinically meaningful endpoints, extended follow-up periods, and more diverse patient populations. Such studies are essential to establish definitive therapeutic value, determine treatment durability, and support evidence-based clinical recommendations.

## Conclusions

Our research demonstrates that non-cross-linked HA injections represent a safe and effective option for skin biorevitalization in real-world aesthetic practice within the defined target population. These findings provide aesthetic practitioners with essential knowledge for making informed clinical decisions and setting realistic expectations regarding the performance and safety of non-cross-linked HA-based medical devices, ultimately improving patient care. Future studies in broader, less restrictive cohorts are warranted to enhance generalizability.
